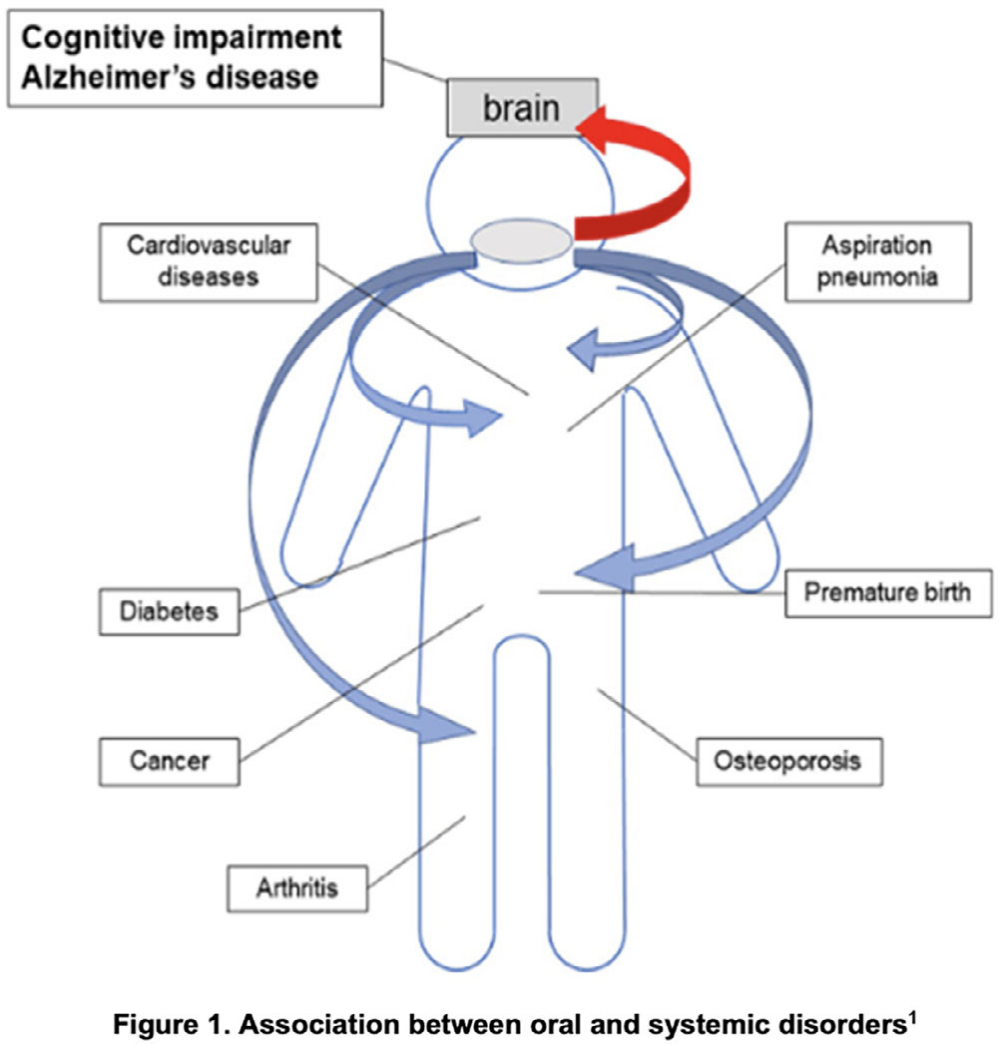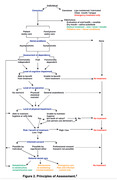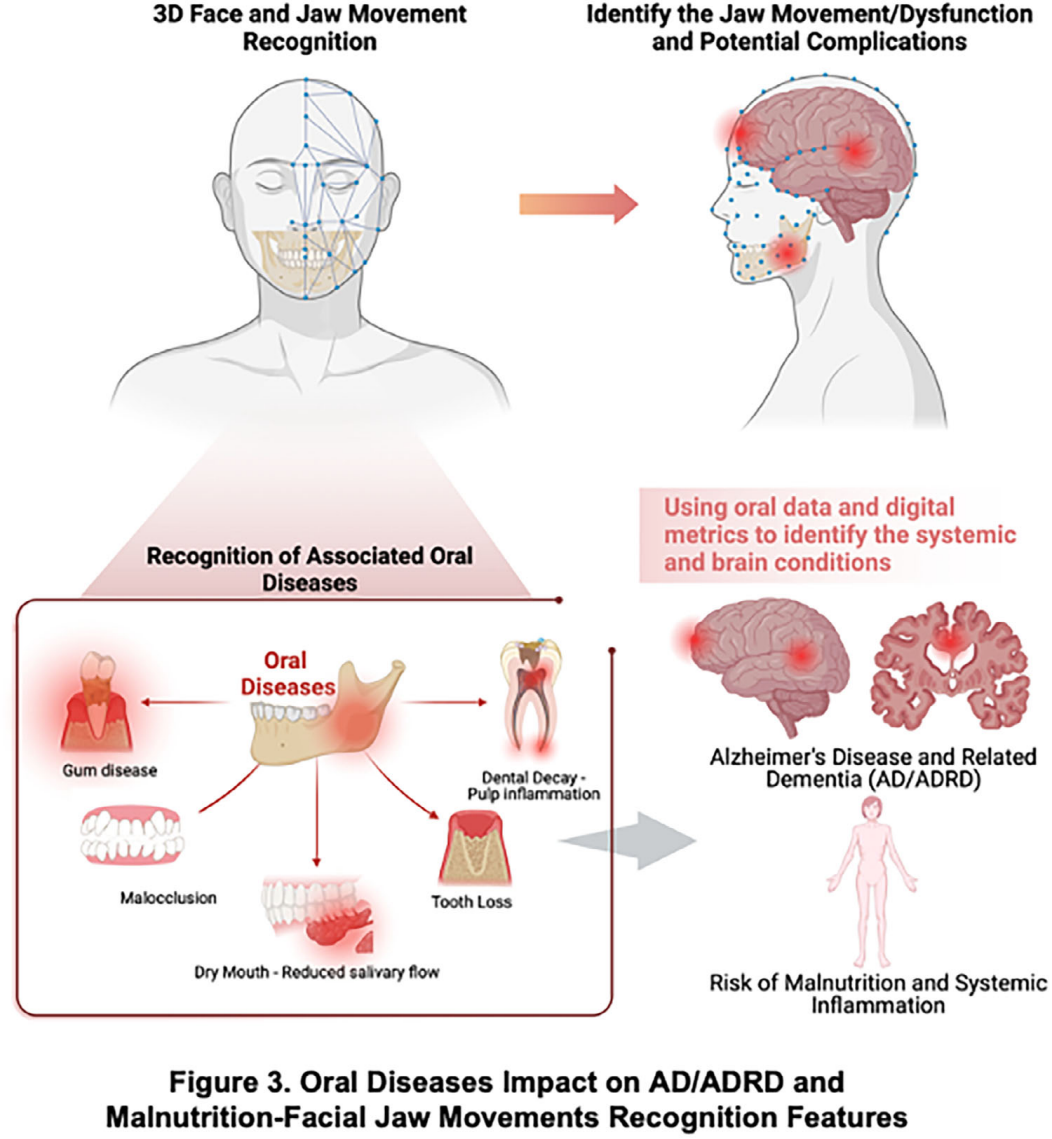# Bridging Oral Health and Alzheimer’s Risk: The “Mouth to Mind” Project

**DOI:** 10.1002/alz.089640

**Published:** 2025-01-09

**Authors:** Steffany Chamut, Bruce A. Dye, Akwaugo Igwe, Spencer Low, Salman Rahman, Hamed Tabkhi, Rhoda Au

**Affiliations:** ^1^ Harvard School of Dental Medicine, Boston, MA USA; ^2^ School of Dental Medicine. University of Colorado Anschutz Medical Campus, Aurora, CO USA; ^3^ Boston University Chobanian & Avedisian School of Medicine, Boston, MA USA; ^4^ Boston University School of Public Health, Boston, MA USA; ^5^ Davos Alzheimer’s Collaborative, Philadelphia, PA USA; ^6^ Boston University School of Medicine, Boston, MA USA; ^7^ Department of Public Health, School of Public Health, Boston University, Boston, MA USA; ^8^ University of North Carolina Charlotte (UNC Charlo, Charlotte, NC USA; ^9^ Alzheimer’s Disease Research Center, Boston University Chobanian & Avedisian School of Medicine, Boston, MA USA; ^10^ Boston University Alzheimer’s Disease Center, Boston University, Boston, MA USA; ^11^ The Framingham Heart Study, Boston University School of Medicine; Boston University School of Public Health, Boston, MA USA; ^12^ Department of Neurology, Boston University Chobanian & Avedisian School of Medicine, Boston, MA USA; ^13^ Boston University Chobanian & Avedisian School of Medicine and School of Public Health, Boston, MA USA; ^14^ Department of Anatomy and Neurobiology, Neurology and Medicine, Framingham Heart Study, BU Alzheimer’s Disease Research Center, Boston University Chobanian & Avedisian School of Medicine, Boston, MA USA

## Abstract

**Background:**

Global health is increasingly burdened by oral diseases (ODs) affecting 3.9 billion people, and Alzheimer’s disease with related dementias (AD/ADRD), impacting 46.8 million globally. Both are expected to increase due to aging populations and greater tooth retention. With soaring dental costs highlighting economic implications and complexities in access to care, there is an urgent need for early diagnosis and preventative low‐cost and accessible interventions. The “Mouth to Mind” project aims to develop and validate a comprehensive oral‐jaw health assessment tool that identifies early signs of cognitive impairment, focusing on the impact of jaw‐occlusal dysfunctions and exploring the intersection of oral health with neurodegenerative risks, malnutrition, and equity.

**Method:**

A questionnaire, part of an oral‐jaw health assessment tool, is being validated with a cohort of participants aged 40 and above (n = 23) who are part of a larger study providing feedback on different health technologies. Qualitative analyses are being used to evaluate the tool’s feasibility and clinical utility. Key outcomes include participant burden, measured by questionnaire completion time, and suggestions for improving questions and answers to enhance clarity and usability.

**Results:**

The study presents 20 females and 3 Males across diverse ethnic and racial groups (n = 8 Whites; n = 4 Hispanics; and n = 11 Black or African American) from the Greater Boston area. Anticipated results will be supportive of a validated questionnaire with insights into its administration, informing the design of a non‐invasive, user‐friendly tool for non‐dental settings.

**Conclusions:**

This study lays the groundwork for the “Mouth to Mind” tool, aimed at early detection of oral diseases and jaw‐occlusal dysfunctions. By integrating oral health into the broader spectrum, it addresses cognitive decline risks. The next phase involves linking jaw‐occlusal dysfunction data with malnutrition and AD/ADRD risks, fostering the development of a transformative digital oral‐cognitive health tool. This tool is expected to advance early detection and preventive care, enhance life quality across populations, and drive significant policy and economic improvements in healthcare management.